# Research on Energy Management of Hybrid Unmanned Aerial Vehicles to Improve Energy-Saving and Emission Reduction Performance

**DOI:** 10.3390/ijerph17082917

**Published:** 2020-04-23

**Authors:** Mingliang Bai, Wenjiang Yang, Dongbin Song, Marek Kosuda, Stanislav Szabo, Pavol Lipovsky, Afshar Kasaei

**Affiliations:** 1 School of Astronautics, Beihang University, Beijing 100191, China; baiml@buaa.edu.cn (M.B.); buaasdb@163.com (D.S.); Akasaei@alum.sharif.edu (A.K.); 2 Faculty of Aeronautics, Technical University of Kosice, 04121 Kosice, Slovakia; marek.kosuda@tuke.sk (M.K.); stanislav.szabo@tuke.sk (S.S.); pavol.lipovsky@tuke.sk (P.L.)

**Keywords:** energy management, hybrid power system, UAV, energy-saving

## Abstract

The rapid development of industry results in large energy consumption and a negative impact on the environment. Pollution of the environment caused by conventional energy sources such as petrol leads to increased demand for propulsion systems with higher efficiency and capable of energy-saving and emission reduction. The usage of hybrid technology is expected to improve energy conversion efficiency, reduce energy consumption and environmental pollution. In this paper, the simulation platform for the hybrid unmanned aerial vehicle (UAV) has been built by establishing the subsystem models of the UAV power system. Under the two chosen working conditions, the conventional cruise flight mission and the terrain tracking mission, the power tracking control and Q-Learning method have been used to design the energy management controller for the hybrid UAV. The fuel consumption and pollutant emissions under each working condition were calculated. The results show that the hybrid system can improve the efficiency of the UAV system, reduce the fuel consumption of the UAV, and so reduce the emissions of CO_2_, NO_x_, and other pollutants. This contributes to improving of environmental quality, energy-saving, and emission reduction, thereby contributing to the sustainable development of aviation.

## 1. Introduction

The rapid development of the aviation industry not only brings great social progress and economic benefits, but also brings increasing contentious environmental pollution problems. It has become a common goal for the development of the aviation industry to ensure the development and protection of the environment on which human beings depend [1,2]. The growing demand for environmental protection has pushed the aviation industry onto a one-way green aviation [3], and technological innovation to meet this demand will lead to a series of major changes in the industry [4]. The fuel used in conventional aircrafts is mostly the aviation kerosene, which mainly emits carbon dioxide (CO_2_), nitrogen oxides (NO_x_), hydrocarbons (C_x_H_y_), carbon monoxide (CO), and other pollutants [5,6]. The main environmental impacts of these pollutants are: leading to the greenhouse effect of the atmosphere, which in turn affects global climate change; reduction of stratospheric ozone, resulting in increased surface ultraviolet radiation [7,8,9]. In addition, the NO_x_ emitted by aircraft can change the composition of chemical molecules in the atmosphere [10]. Subsequently, many studies report association between exposure to air pollution and the risk of diseases such as chronic and acute respiratory, lung cancer, cardiovascular diseases, and premature deaths [11,12,13,14,15,16]. Short-term exposure to traffic-related air pollution shows association with adverse cardiovascular problems [17,18]. An increased risk of several types of cancers has been link to the working environment of aircraft crew with increased levels of known or suspected carcinogenic elements such as engine emissions, poor air quality, and ionizing radiation [19]. Neurodegenerative diseases as well as the potential to cause type-2 diabetes and leukemia were documented to be potential effects of air pollution [20,21]. Combination of traffic-related air and noise pollution was examined and it was shown that exposure to both may result in cardiac autonomic dysfunction where the effect of air pollutants is amplified at a high noise level [22]. Moreover, air pollution was associated with an increase in the number of hospitalizations due to chronic obstructive pulmonary disease in European cities with versatile climates [23,24]. The need for reduction in consumption of the conventional aviation fuel is therefore one of the key issues being addressed at the national level as it leads to lessening overall impact on the environment and on public health.

More attention has been paid to environmental problems of air traffic, the United States and the European Union [25] have put forward new requirements for the next generation of commercial aircrafts, focusing on fuel consumption, noise control, pollution emissions and other aspects, and have formulated specific development objectives [26]. National Aeronautics and Space Administration (NASA) proposed a performance target [27] of reducing fuel consumption, emissions reduction, and low noise for subsonic airliners in 2008, which is divided into three phases: phase 1 to 2015 (n + 1), phase 2 to 2020 (n + 2), and phase 3 to 2030 (n + 3). The European Union has also set a performance target of subsonic aircraft in the future. It plans to achieve a 75% reduction in carbon dioxide emissions, 90% reduction in nitrogen oxide emissions, and 65% reduction in noise emissions by 2050 based on the technical level in 2000 [28].

The technical progress of the aircraft power system is a necessary guarantee for further improving fuel efficiency and reducing emissions. Hybrid propulsion technology is an important development direction of aviation propulsion technology in the future [29]. By integrating the advantages of multi-energy systems, hybrid propulsion technology is expected to further improve energy conversion efficiency and reduce aircraft fuel consumption, achieve the goal of energy-saving, reduce emission, and environmental protection [30,31]. The purely electric propulsion system is composed of the power supply system and electric propulsion system and does not need an engine. Pure electric aircraft has the advantage of zero emission during flight, and the noise level during the aircraft operation is often very low [32]. However, due to limited accumulators technology, the load, flight speed, flight range, and flight duration of this type of aircraft are limited; it is difficult for large airliners to use them at present [33].

A research team at the University of Cambridge in the UK developed the world’s first hybrid aircraft in 2012, which uses a parallel hybrid system equipped with a piston engine and an electric motor. During the take-off phase, the engine and the motor work at the same time to meet the power requirements for takeoff; when the cruising altitude is reached, the motor switches to the power generation mode, charges the accumulator, or switches to engine assist mode to save fuel. The aircraft can save about 30% more fuel compared to a similar aircraft [34]. The Boeing ‘SUGAR Volt’ hybrid aircraft is equipped with the ‘hfan’ hybrid power plant developed by GE. The motor is installed directly in the engine and the parallel petrol–electric hybrid propulsion system is used as its power scheme—the fuel consumption is reduced by 28% compared with the CFM56 turbofan engine [35,36].

Regarding unmanned aerial vehicles (UAVs), it is wise to consider an overall scheme of the hybrid power system based on the existing electromotors and aviation internal combustion engines technology. This combination maintains the efficient characteristics of electromotors, but also uses traditional internal combustion engines to reduce the in-flight fuel consumption and increase the flight range, which has become an urgent problem to be solved in hybrid power technology. According to the specific flight path of the UAV itself, the energy management controller [37] can dynamically distribute the output power of motor and internal combustion engine in real time to meet the power demands of the UAV [38]. This is based on the assumption of considering the accumulator charge and discharge power, loss and the output torque, speed and other constraints of internal combustion engine [39]. Energy management algorithms for hybrid power systems are mainly divided into rule-based control algorithms and optimization-based control algorithms [40,41]. The novel control strategies using reinforced and predictive learning to outweigh the negative characteristics of the model predictive control or the equivalent consumption minimization strategy (ECMS), which require elaborate vehicle models, showed positive results in improved fuel economy [42,43]. In the case of hybrid electric vehicles, thermostat control strategy (TCS) and power tracking control strategy (PTCS) represent the most conventional ruled-based controllers, but they do not consider fuel economy optimization. The exclusive operation strategy, which combines the most effective design principles of TCS and PTCS while implementing ECMS improved fuel economy, but still falls behind in comparison to optimization-based benchmarks [44]. Kong et al. further improved fuel economy of hybrid tracked vehicles, a real-time reinforced learning control strategy using the recursive algorithm to compute the transitional probability matrix showed a decrease of fuel consumption in comparison to the stationary strategy [45]. In a nutshell, rule-based algorithms rely on actual engineering experience and are now widely used in automotive energy management systems [46] and optimization-based algorithms use analytic or numerical methods to reduce the objective function taking in consideration of optimization objectives [47].

The following paper presents a study of energy management of the petrol–electric hybrid propulsion system for the hybrid fixed-wing UAV. The goal is for the hybrid propulsion system to save energy and reduce emissions and improve the aviation impact on the environment. The subsystem model of the UAV hybrid power system has been established, and the UAV simulation platform was built. The energy management controller of the hybrid UAV was designed by using power tracking control and Q-Learning control methods, it was simulated and analyzed under two operating conditions: conventional cruise mission and terrain tracking mission, which can provide a theoretical reference for improvement of the energy conversion efficiency of UAV systems and reduce fuel consumption.

## 2. Establishment of UAV Subsystem Model and Simulation Platform

**Figure 1:**
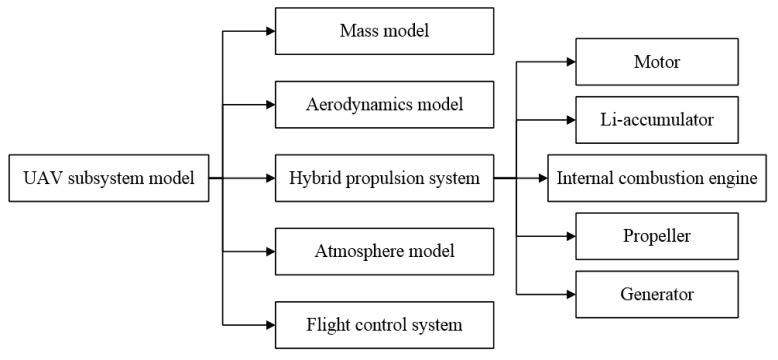
The model of hybrid the unmanned aerial vehicle (UAV) subsystem.

Subsystem modeling is an important foundation for UAV energy management simulation and optimization research. The mathematical model of the sub-system of the UAV and the construction of the simulation platform was performed in Matlab/Simulink software. Hybrid propulsion system modeling is mainly divided into five modules: generator, lithium accumulator, internal combustion engine, propeller, and energy management controller. Other implemented models such as mass, flight control system, and standard follow conventional models and standards, therefore they are not separately introduced. The various subsystem models required for the UAV energy management simulation and optimization institute are shown in Figure 1.

### 2.1. Flight Dynamics Model

#### 2.1.1. Aerodynamic Force and Aerodynamic Moment

The main forces on the UAV are propeller thrust, lift, drag, gravity, etc. The aerodynamic force of the UAV in the direction of three orthogonal axes in the body coordinate system is:

(1) $$F_{aero}=\left[\begin{array}{c} F_{aeroD}\\ F_{aeroY}\\ F_{aeroL} \end{array}\right]=\frac{1}{2}\rho V^{2}S\;\left[\begin{array}{c} C_{D}\\ C_{Y}\\ C_{L} \end{array}\right],$$

where $C_{D}$, $C_{Y}$, and $C_{L}$ are aerodynamic coefficients in drag, sideslip, lift force directions.

The aerodynamic torque is:

(2) $$M_{p}=\left[\begin{array}{c} L_{aeroX}\\ L_{aeroY}\\ L_{aeroZ} \end{array}\right]=-\frac{1}{2}\rho V^{2}S\;\left[\begin{array}{c} C_{l}\\ C_{m}\\ C_{n} \end{array}\right],$$

where $C_{l}$, $C_{m}$, and $C_{n}$ are aerodynamic coefficients in rolling, pitching, yaw moment directions.

Lift coefficient $C_{L}$, drag coefficient $C_{D}$, sideslip force coefficient $C_{Y}$, pitching moment coefficient $C_{m}$, rolling moment coefficient $C_{l}$, and yaw moment coefficient $C_{n}$ are defined respectively as illustrated in [48].

#### 2.1.2. Kinetic and Kinematic Equations

Defining the speed of the UAV in this system is expressed as [u, v, w], then the acceleration is:

(3) $$\{\begin{array}{l} \dot{u}=X/m+g_{x}+rv-qw\\ \dot{v}=Y/m+g_{y}-ru-pw\\ \dot{w}=\frac{Z}{m}+g_{z}+qu-pv \end{array},$$

where *m* is the total mass of the UAV, [p, q, r] is the pitch, roll, and yaw rate of the UAV in this system.

The position of the UAV in the inertial system can be expressed as $\left[\begin{array}{ccc} x_{I} & y_{I} & z_{I} \end{array}\right]$, and the speed in the inertial system is:

(4) $$\{\begin{array}{l} {\dot{x}}_{I}=\left(cos\theta cos\psi \right)u+\left(sin\phi sin\theta cos\psi -cos\phi sin\psi \right)v+\left(sin\phi sin\psi +cos\phi sin\theta cos\psi \right)w\\ {\dot{y}}_{I}=\left(cos\theta sin\psi \right)u+\left(sin\phi sin\theta sin\psi +cos\phi cos\psi \right)v+\left(-sin\phi cos\psi +cos\phi sin\theta sin\psi \right)w\\ {\dot{z}}_{I}=\left(-sin\theta \right)u+\left(sin\phi cos\theta \right)v+\left(cos\phi cos\theta \right)w \end{array},$$

where $\left[\begin{array}{ccc} \phi & \theta & \psi \end{array}\right]$ is the vector of Euler attitude angles of pitch, roll, and yaw. Then the angular acceleration is:

(5) $$\{\begin{array}{l} \dot{p}=(I_{zz}L+I_{xz}N-\{I_{xz}\left(I_{yy}-I_{xx}-I_{zz}\right)p+\left[I_{xz}^{2}+I_{zz}\left(I_{zz}-I_{yy}\right)\right]r\}q)/\left(I_{xx}I_{zz}-I_{xz}^{2}\right)\\ \dot{q}=\left[M-\left(I_{xx}-I_{zz}\right)pr-I_{xz}\left(p^{2}-r^{2}\right)\right]/I_{yy}\\ \dot{r}=(I_{xz}L+I_{xx}N+\{I_{xz}\left(I_{yy}-I_{xx}-I_{zz}\right)r+\left[I_{xz}^{2}+I_{xx}\left(I_{xx}-I_{yy}\right)\right]p\}q)/\left(I_{xx}I_{zz}-I_{xz}^{2}\right) \end{array},$$

where $\left[\begin{array}{ccc} I_{xx} & I_{yy} & I_{zz} \end{array}\right]$ is the main inertia, and $\left[\begin{array}{ccc} I_{xy} & I_{xz} & I_{yz} \end{array}\right]$ is the product of inertia.

The angular velocity in the inertial system is:

(6) $$\{\begin{array}{l} \dot{\phi }=p+\left(qsin\phi +rcos\phi \right)tan\theta \\ \dot{\theta }=qcos\theta -rsin\phi \\ \dot{\psi }=\left(qsin\phi +rcos\phi \right)sec\theta \end{array}.$$

The above differential Equations (1)–(6) can solve the state variables such as flight position, speed, attitude angle, and angular velocity of the UAV.

### 2.2. Hybrid Propulsion System Model

#### 2.2.1. The Model of Internal Combustion Engine and Generator

The internal combustion engine in the hybrid system drives the generator to generate electricity, powers the electromotor, or charges the lithium accumulator. In order to operate the motor at the right speed, a transmission is required between the two devices. The output voltage of the generator is:

(7) $$U_{g}=K_{e}n_{eng}i_{eg}-K_{x}n_{eng}i_{eg}I_{g},$$

where $U_{g}$ and $I_{g}$ are the output voltage and current of the generator, $n_{eng}$ is the rotational speed of the internal combustion engine, $i_{eg}$ is the transmission ratio between the internal combustion engine and the motor, and $K_{e}$ is the electromotive force coefficient. $K_{x}$ is the equivalent impedance coefficient $\frac{K_{x}=3PL^{g}}{\pi }$, P is the number of poles and $L^{g}$ is the armature synchronous inductor of the generator. The relationship between the torque $T_{g}$ of the generator and the current $I_{g}$ is:

(8) $$K_{e}I_{g}-K_{x}I_{g}^{2}=T_{g}.$$

Due to the gearbox connection, the speed relationship between the internal combustion engine and the generator is:

(9) $$n_{g}=n_{en}\cdot i_{eg}.$$

The dynamics model of internal combustion engines and generators is:

(10) $$\left(\frac{J_{e}}{i_{eg}{}^{2}}+J_{g}\right)\frac{d_{n_{g}}}{d_{t}}=\frac{\left(\frac{T_{eng}}{i_{eg}}-T_{g}\right)}{0.1047}$$

where $J_{e}$ and $J_{g}$ are the moments of inertia of the internal combustion engine and the generator respectively. $T_{eng}$ represents the torque of the engine. The fuel consumption of internal combustion engine is defined as:

(11) $$Fuel=\int_{t_{0}}^{t_{f}}{\dot{m}}_{f}\left(n_{en},T_{en}\right)dt,$$

where ${\dot{m}}_{f}\left(n_{en},T_{en}\right)$ is the fuel consumption rate of the internal combustion engine, a function of the speed and torque, which can usually be obtained by using the look-up table method.

**Figure 2:**
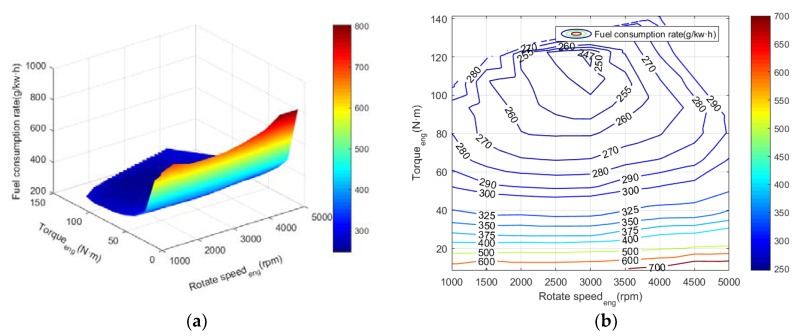
The fuel consumption rate chart of an internal combustion engine in the hybrid system. (**a**) Cloud map; (**b**) Contour map.

**Figure 3:**
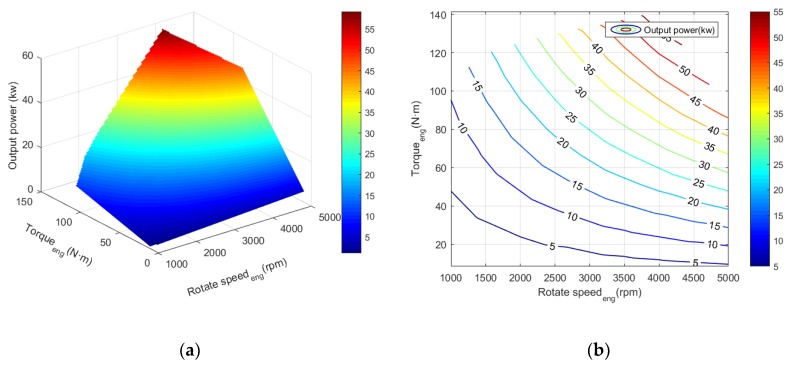
The power diagram of internal combustion engine in the hybrid system. (**a**) Cloud map; (**b**) Contour map.

The operating characteristics curve of the internal combustion engine is shown in Figure 2 and Figure 3, wherein both the engine power and the fuel consumption rate can be interpolated from the rotational speed and the torque. Figure 2 is the fuel consumption rate data of the internal combustion engine, and Figure 3 corresponds to the data of the engine power. The maximum power of an internal combustion engine is approximately 70 kW. From the fuel consumption rate curve, the fuel consumption rate of the internal combustion engine is the lowest in the area adjacent to the rotation speed of 3000 rpm and the torque of 110 N·m, where the power of the internal combustion engine is about 30 kW. The upper bound of the contour line is called the external characteristic of the internal combustion engine, which is the curve connected by the maximum point of output torque and output power achieved by the internal combustion engine. In the process of simulation, the operating points of the engine should all be located inside the external characteristic curve.

#### 2.2.2. The Model of Li-Accumulator

Ignoring the effect of temperature on the operating state of the accumulator, Li-accumulator can be modeled using an internal resistance model [49]. The differential of the state of charge of the accumulator is defined as:

(12) $$\dot{SOC}=-I_{accu}\left(t\right)/C_{accu},$$

where $I_{bat}\left(t\right)$ is the current of the accumulator at t time and $C_{accu}$ is the capacity of the accumulator. Generally, the SOC of Li-accumulator is an important state variable in energy management. The internal resistance of the accumulator varies with the charging and discharging, so the output voltage of the accumulator is also divided into two cases: charging and discharging:

(13) $$U_{accu}=\{\begin{array}{l} V\left(SOC\right)-I_{accu}\cdot (R_{i_{ch}}\left(SOC\right)+R_{t})(I_{accu}>0)\\ V\left(SOC\right)-I_{accu}\cdot (R_{i_{dis}}\left(SOC\right)+R_{t})\left(I_{accu}\leq 0\right) \end{array},$$

where $R_{t}$ is the terminating resistor, $R_{i_{ch}}$and $R_{i_{dis}}$ are the internal resistances in the case of accumulator charging and discharging, and can all be represented by $R_{int}\left(SOC\right)$, and V(SOC) is the open circuit voltage of Li-accumulator. The output power of the accumulator pack is:

(14) $$P_{accu}=U_{accu}\cdot I_{accu},$$

where $\dot{SOC}$ can be derived from Equations (19)–(21):

(15) $$\dot{SOC}=\frac{\left(V\left(SOC\right)-\sqrt{V^{2}\left(SOC\right)-4(R_{int}\left(SOC\right)+R_{t})P_{accu}\left(t\right)}\right)}{2C_{accu}(R_{int}\left(SOC\right)+R_{t})}.$$

#### 2.2.3. The Model of Motor and Propeller

**Figure 4:**
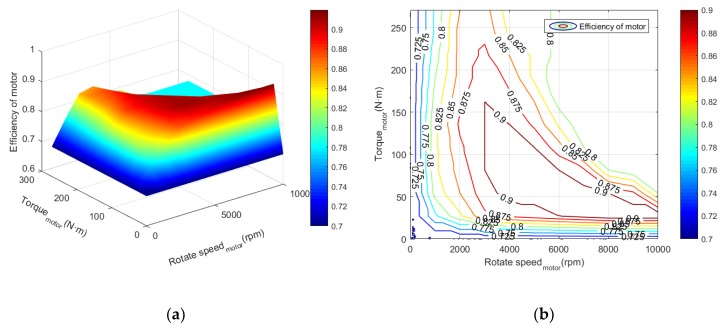
The efficiency diagram of drive motor in hybrid system. (**a**) Cloud map; (**b**) Contour map.

The permanent magnet synchronous motor (PMSM) is used as the driving motor in this study. According to the principle of power conservation, the power of the input motor is equal to the output mechanical power of the motor and the heat loss power of the motor:

(16) $$U_{m}\cdot I_{m}\cdot \eta_{em}=T_{m}\cdot \omega_{m},$$

where $U_{m}$, $I_{m}$, $\eta_{em}$, $T_{m},$ and $\omega_{m}$ represent the input voltage, current, efficiency of the motor, output torque, and rotational speed of the rotor respectively. $\eta_{em}$ is a function of the motor speed and torque, and generally it can be measured experimentally. The efficiency can be obtained by interpolation of data in Figure 4.

The real-time power needed for UAV flight is provided by both the accumulator and the internal combustion engine to meet the power balance:

(17) $$P_{req}=\left(U_{g}\cdot I_{g}\cdot \eta_{g}+U_{accu}\cdot I_{accu}\right)\cdot \eta_{em},$$

where $\eta_{g}$ represents the rectifier efficiency of generator.

The UAV adopts fixed pitch propeller, and its advance coefficient is:

(18) $$J=\frac{\pi V_{a}}{\Omega R},$$

where R is the radius of the propeller and $V_{a}$ is the airspeed. The thrust and moment of the propeller are:

(19) $$\{\begin{array}{c} F_{p}=\frac{4\rho R^{4}\Omega^{2}C_{T}}{\pi^{2}}\\ M_{p}=\frac{—4\rho R^{5}\Omega^{2}C_{P}}{\pi^{3}} \end{array}$$

where $C_{T}$ is the thrust coefficient, $C_{P}$ is the power coefficient, Ω is the propeller speed, and ρ is the current air density of the UAV provided by the standard atmospheric model.

## 3. Flight Simulation Calculation Flow of UAV

**Figure 5:**
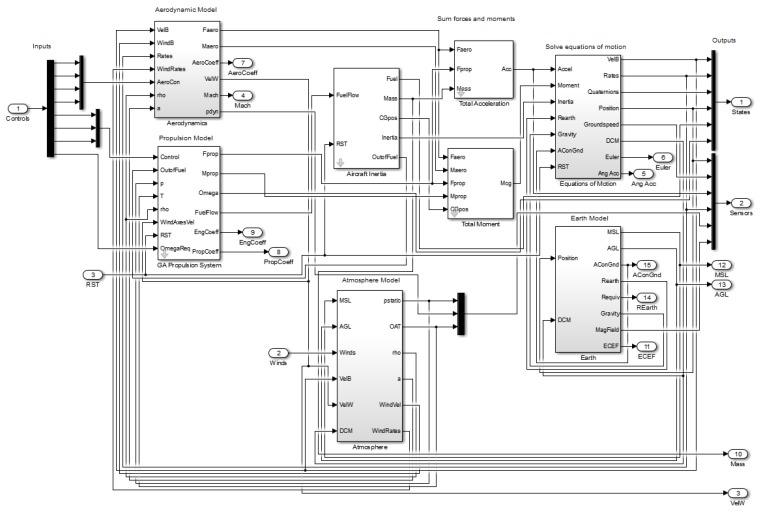
The Simulink model of the UAV subsystem.

Table 1 shows the basic parameters of the flight simulation UAV used in this study. Flight simulation calculation includes the process of the loading UAV mission curve, flight control, calculation of required power, calculation of fuel consumption rate of hybrid power system, mass of the whole aircraft, and so on. In the flight simulation mission, the upper flight control system is used to control the UAV to fly according to a given mission curve, and the power demand of the whole machine is calculated and transmitted to the lower energy management controller. This forms a closed loop control to meet the UAV power requirements in real time. The real-time demand power of the UAV is determined by the output power of the propeller. Due to the energy loss caused by the conversion of the multi-stage power in the system, it is necessary to consider the working efficiency of the motor, and finally convert the required power of the propeller into the required power of the hybrid system. The power controller controls the generator and the lithium accumulator to provide sufficient power. Due to the continuous consumption of fuel during the flight, the weight of the whole machine will change. The fuel consumption data is collected in real time by the hybrid system and fed back to the quality model calculation module of the whole machine, so that the fuel consumption of the entire task can be calculated more accurately. The Simulink model of the UAV subsystem simulation is shown in Figure 5.

**Figure 6:**
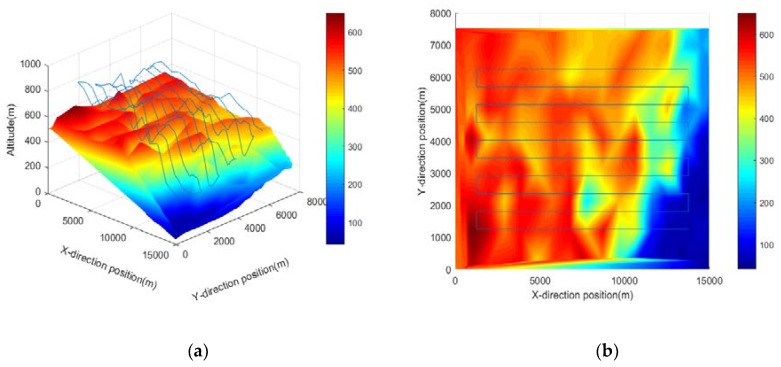
Hybrid UAV terrain tracking mission curve. (**a**) Three-dimensional diagram; (**b**) Planar graph.

A typical flight profile curve needs to be selected to calculate the required power for different tasks. Two typical operating conditions were selected: conventional cruise flight and terrain tracking flight. Conventional cruise flight includes three stages: take-off climb, constant altitude flight, and descent to landing. The task is simple and the change of power demand is smooth. In the terrain tracking mission, the UAV needs to track the terrain according to the planned mission route while maintaining a certain altitude flight with the ground. Terrain tracking is often used for remote sensing imaging, mapping, or evading ground radar detection by UAVs carrying optoelectronic pods. As shown in Figure 6, the UAV performs a “Z”-shaped flight in the X direction and advances stepwise in the Y direction to form a mission profile curve. In contrast, in the terrain tracking mission, the flight altitude of the UAV changes frequently, and the corresponding demand power also changes as the UAV climbs or falls. Therefore, the two mission profile curves can calculate two typical operating conditions: demand power is stable and demanded power is frequently changed.

**Figure 7:**
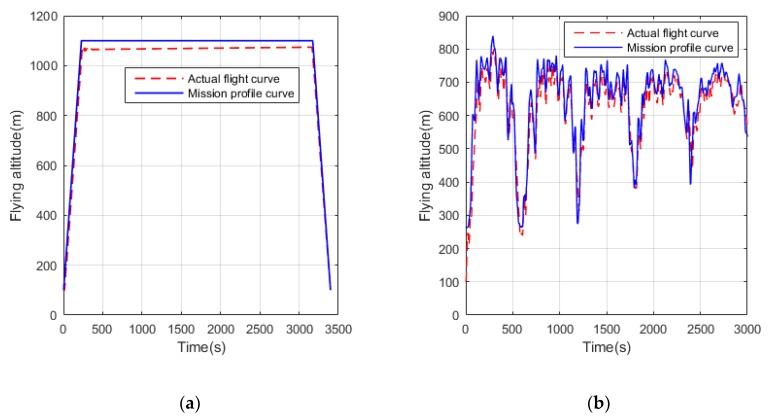
The actual flight trajectory and mission profile curve of the UAV. (**a**) Conventional cruise mission; (**b**) Terrain tracking mission.

**Figure 8:**
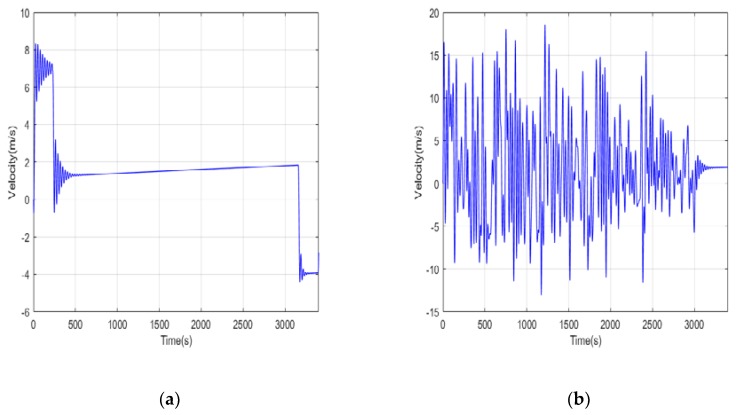
The speed curve of UAV flight. (**a**) Conventional cruise flight mission; (**b**) Terrain tracking flight mission.

**Figure 9:**
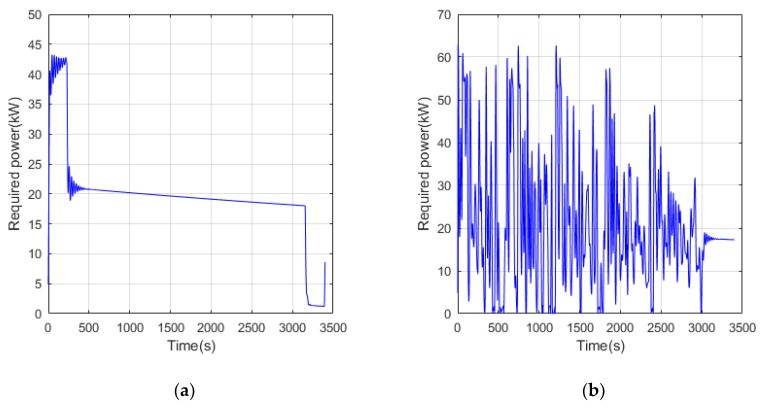
The required power curve of UAV flight. (**a**) Conventional cruise flight mission; (**b**) Terrain tracking flight mission.

It is necessary to use a flight trajectory tracker to track the position in the mission profile in real time during a real flight mission. In order to simplify the task, only the height is used as the tracking target and the actual flight curve is shown in Figure 7. In most of the flight time, the actual flight curves of the UAVs under two different operating conditions are close to the task profile curve, which verifies the effect of the trajectory tracker. The Z-direction speed curve and demanded power of the UAVs are shown in Figure 8 and Figure 9, respectively. It can be seen that in the conventional cruise mission, the required power fluctuates only during the transitional state of the trajectory tracker. In the terrain tracking mission, the demand power changes more severely because of the need to track the changing altitude constantly.

According to the above calculation process, given the flight profile curve of the UAV, the corresponding power demand curve can be calculated, which can provide a basis for analyzing the dynamic characteristics of the power demand and solving the probability transfer matrix. The demanded power of the UAV is related to the task profile curve. In many tasks, the task profile curve can be determined before the flight starts, so the results obtained based on the optimized energy management strategy are similar to the results in the actual flight process.

## 4. Energy Management of UAV Hybrid System

### 4.1. Energy Management Model of Hybrid Power System

The controlled object is the hybrid power system and the control strategy presents the energy management control algorithm in the energy management of the UAV. The core task of applying reinforcement learning to UAV energy management is to get the optimal controller through the iterative optimization of the reinforcement learning algorithm to minimize the fuel consumption during flight. Therefore, the optimization objective function is defined as:

(20) $$J=\int_{0}^{T}\left[f_{rate}\left(t\right)+\alpha \Delta_{SOC}^{2}\right]\;d_{t}$$

where $f_{rate}\left(t\right)$ is the fuel consumption rate at time t. $\Delta_{SOC}$ is the accumulator SOC terminal constraint, its expression is:

(21) $$\Delta_{SOC}=\{\begin{array}{c} SOC\left(t\right)-SOC_{ref},SOC\left(t\right)>SOC_{ref}\\ 0,SOC\left(t\right)<SOC_{ref} \end{array}$$

where $SOC_{ref}$ is the set SOC reference value. The purpose of terminal constraints is to limit state of charge (SOC) to the reference value as much as possible, so as to achieve the purpose of charge discharge balance. α is the weight coefficient between fuel consumption and accumulator loss. The physical meaning of the objective function is to minimize the fuel consumption rate and accumulator loss.

In order to ensure the safety of the power and energy systems, the following optimization constraints shall be met for the simulation:

(22) $$\{\begin{array}{c} \begin{array}{c} 0.4\leq SOC\left(t\right)\leq 0.9\\ 0\leq T_{eng}\left(t\right)\leq 150\left(N\cdot m\right) \end{array}\\ \begin{array}{l} -10\leq P_{bat}\left(t\right)\leq 30\left(kW\right)\\ 0\leq n_{eng}\leq 5000\left(r/min\right) \end{array} \end{array}$$

The physical meanings of the above inequality constraints are as follows:

- Ensure that the state of charge and discharge of the Li-accumulator is in a reasonable range, the maximum value of *SOC* is 0.9; the minimum value of SOC is 0.4, ensuring that the accumulator will not be damaged by excessive discharge.
- Ensure that the engine torque is in a reasonable range;
- Ensure that the accumulator charging and discharging power is in a reasonable range, if the accumulator power is negative, it represents the accumulator charging;
- Ensure that the engine speed is in a reasonable range, where the working point with zero speed represents the engine shutdown, and the torque at this working point is also zero.

### 4.2. Design of Energy Management Controller

In the hybrid propulsion system of the UAV, the energy management controller is its core module. Different forms of energy sources need to be scheduled by the energy management controller to effectively provide thrust. The energy management controller of the hybrid system has the following three functions. First of all, according to the real-time power demand of the UAV, the controller schedules the power distribution ratio between the internal combustion engine and the lithium accumulator, so as to make the internal combustion engine work in the high efficiency range as much as possible to save fuel. In addition, the energy management controller can also achieve the purpose of protecting system components by monitoring the real-time operation parameters of each subsystem and adjusting the operation status of the system. Finally, through the additional energy storage system (such as Li-accumulator), in a certain period of time, the hybrid propulsion system can output more power than the traditional internal combustion engine propulsion system, to meet the demand of short-term peak power. The Q-Learning method in reinforcement learning and power tracking control is used to design the energy management controller respectively.

#### 4.2.1. Power Tracking Control

**Figure 10:**
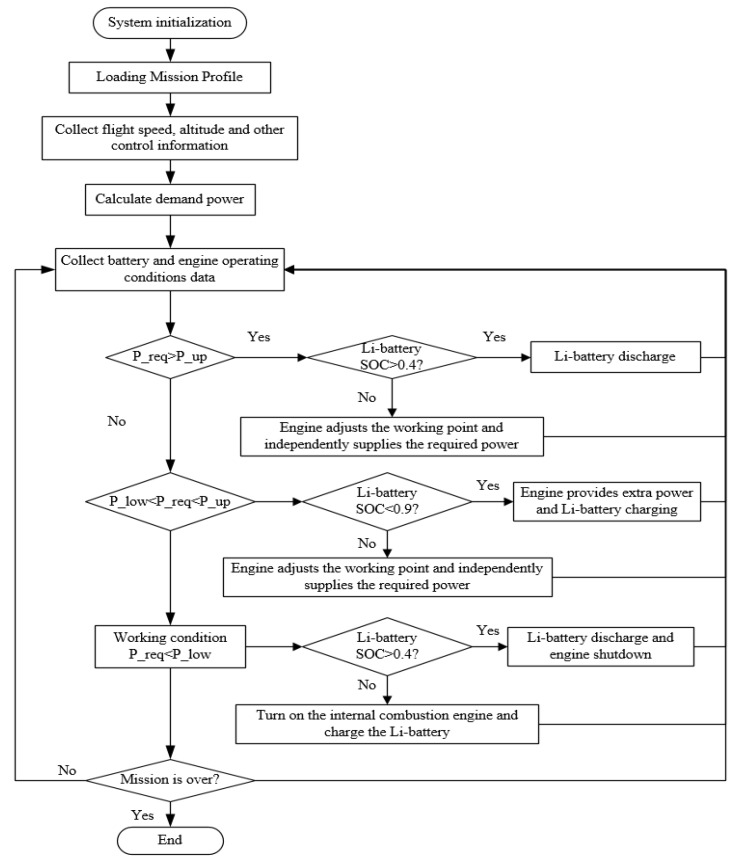
The flow chart of the power tracking control.

The characteristic behavior of the power tracking control is that the internal combustion engine tracks the power demand of the UAV in real time, and only idle or shut down when the accumulator pack reaches the maximum charging state and can meet the power meter requirements of the UAV. The power tracking control covers the limited combination of the internal combustion engine and lithium accumulator, and the required power and its control flow chart is shown in Figure 10.

The presented design always tries to keep the internal combustion engine working in the high efficiency range. When the power of the internal combustion engine is not sufficient to meet the required power, the accumulator is used as the auxiliary power supply to avoid the internal combustion engine working in the high-power rate and high fuel consumption area. When the demanded power is very low and the internal combustion engine needs to work in the low power and high fuel consumption area, the accumulator is used to supply power independently and the internal combustion engine is shut down to save fuel. On the contrary, if the accumulator fails to supply power, the internal combustion engine will start up and work in the high efficiency range and charge the accumulator at the same time, so as to work in this way of cyclic charging and discharging.

#### 4.2.2. Q-Learning Method

In the energy management of the UAV, it is necessary to study the demand power of the UAV and the dynamic characteristics in the process of demand power change. Furthermore, the state transition probability matrix can be extracted from the demanded power curve. The transfer probability matrix of demand power is used to describe the dynamic characteristics of power demands during flight, which is the basis of using reinforcement learning to solve the optimal control strategy. The required power $P_{req}$ and velocity v of the UAV are divided into the following finite sets:

(23) $$P_{req}\in \left\{P_{req}^{1},P_{req}^{2},\ldots ,P_{req}^{N_{p}}\right\}.$$

(24) $$v\in \left\{v^{1},v^{2},\ldots v^{N_{v}}\right\}.$$

The state transition probability is defined as:

(25) $$p_{ik,j}=\frac{M_{ik,j}}{M_{ik}}\;\left(m_{ik}\neq 0\right),$$

where $M_{ik,j}$ represents the total number of times that the required power is converted from $P_{req}^{i}$ to $P_{req}^{i}$ at the speed of $v_{ave}^{k}$. $M_{ik}$ is the total number of times that the required power $P_{req}^{i}$ occurs at a speed of $v_{ave}^{k}$. The state transition probability shall meet the following conditions:

(26) $$\{\begin{array}{c} M_{ik,j}\geq 0\\ \overset{N_{p}}{\underset{j=1}{\sum }}M_{ik,j}=M_{ik} \end{array}.$$

**Figure 11:**
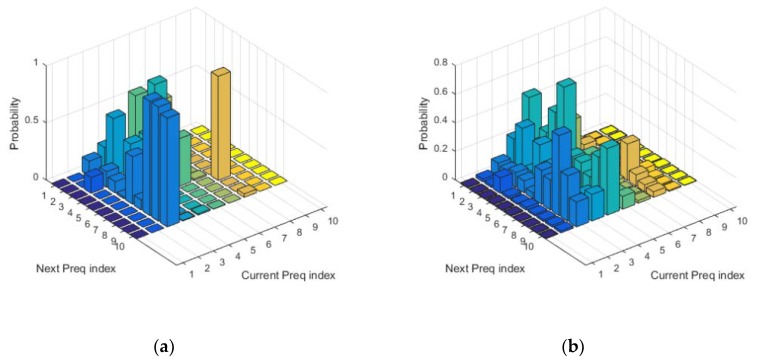
Transfer probability matrix of demand power. (**a**) Under normal conditions; (**b**) After smoothing processing.

The first condition guarantees that the probability is greater than or equal to zero, and the second condition is the normalization condition of probability. The transition probability of most states will be zero due to the limited points that the demanded power curve can cover in the state space. The probability transition matrix of the demanded power is obtained by using the fuzzy vector quantization (FVQ) processing method [50], as shown in Figure 11.

The optimal state value function is defined as the expectation value of minimizing the sum of the finite discounted returns based on the premise of policy π:

(27) $$V^{*}\left(s\right)=\underset{\pi }{\min}E\left(\sum_{t=t_{0}}^{t=t_{f}}\gamma^{t}r_{t}\right),$$

where π is the energy management control strategy, γ∈[0, 1] is the discount coefficient, and $r_{t}$ is the real-time return at the time of t; since the purpose of optimization is to minimize the fuel consumption, the return is defined as the optimization objective function J. From the uniqueness theorem, the optimal state value function can be rewritten in the form of recursion:

(28) $$V^{*}\left(s\right)=\underset{\pi }{\min}\left(r\left(s,a\right)+\gamma \sum_{s^{\prime }\in S}p_{sa,s^{\prime }}V^{*}\left(s^{\prime }\right)\right)\;\forall s\in S.$$

Thus, the optimal control strategy corresponding to the transition from state s to $s^{\prime }$ is as follows:

(29) $$\pi^{*}\left(s\right)=\underset{\pi }{\arg\;\min}\left(r\left(s,a\right)+\gamma \sum_{s^{\prime }\in S}p_{sa,s^{\prime }}V^{*}\left(s^{\prime }\right)\right).$$

The optimal control strategy is obtained by using the Q-Learning algorithm, and the action utility function Q(s,a) is defined as:

(30) $$Q\left(s,a\right)=r\left(s,a\right)+\gamma \sum_{s^{\prime }\in S}p_{sa,s^{\prime }}Q\left(s^{\prime },a^{\prime }\right).$$

The action utility function Q(s,a) represents the corresponding value function of the (s,a). Therefore, the optimal action utility function refers to finding the optimal control strategy and that is the optimal action $a^{*}$ minimizes the action utility function. The optimal action utility function is defined as:

(31) $$Q^{*}\left(s,a\right)=r\left(s,a\right)\gamma \sum_{s^{\prime }\in S}p_{sa,s^{\prime }}+\underset{a^{\prime }}{\min}Q\left(s^{\prime },a^{\prime }\right).$$

**Figure 12:**
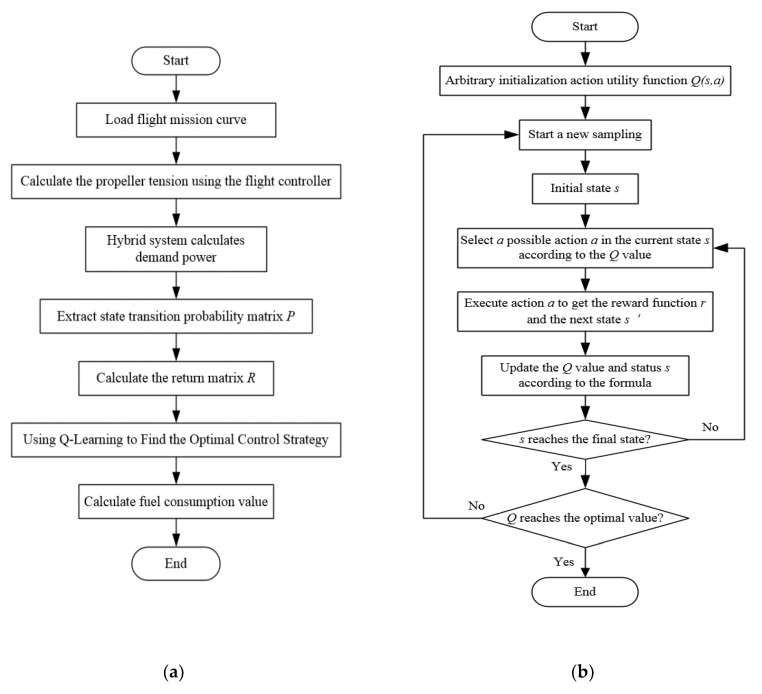
(**a**) The flow chart of solving the optimal control strategy; (**b**) Update process of Q-Learning algorithm.

According to the definition of action utility function Q and value function V, it can be found that the main difference is whether the control quantity a of its initial state is known. The update rules of Q-Learning algorithm are as follows:

(32) $$Q\left(s,a\right)\leftarrow Q\left(s,a\right)+\eta \left((r+\gamma \underset{a^{\prime }}{\min}Q\left(s^{\prime },a^{\prime }\right)-Q\left(s,a\right)\right),$$

where η is the step parameter in the update process, which controls the speed of the convergence process. The state variables of the system are selected as the charge and discharge state *SOC* of the accumulator pack and the required power $P_{req}$ of the UAV, that is $S=SOC,\;P_{req}$. The action of the system is defined as the engine speed ($n_{eng}$) and torque ($T_{eng}$), that is $a=\left[n_{eng},T_{eng}\right]$. The physical meaning of the optimization process is that the controller continuously controls and samples the hybrid power system to get a series of state and action sequences $\left(s_{1},a_{1}\right),\left(s_{2},a_{2}\right),\ldots ,\left(s_{n},a_{n}\right)$ and the corresponding return sequence $r_{1},r_{2},\ldots ,r_{n}$. The control strategy is optimized according to the return, so that the final optimal control sequence can minimize fuel consumption. The flow chart of solving the optimal control strategy and the update process of the Q-Learning algorithm are shown in Figure 12.

### 4.3. Results Analysis and Discussion

#### 4.3.1. Conventional Cruise Flight Mission

**Figure 13:**
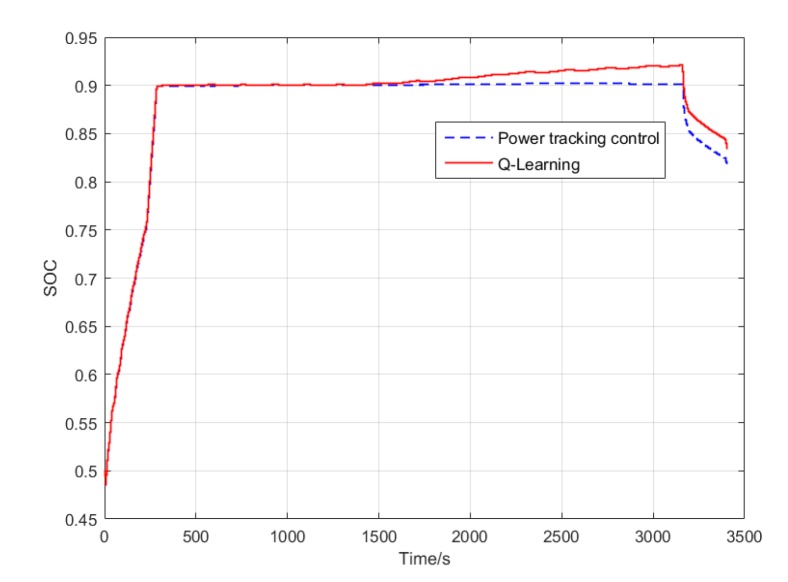
State of charge (SOC) variation curve of two control methods in conventional cruise flight mission.

**Figure 14:**
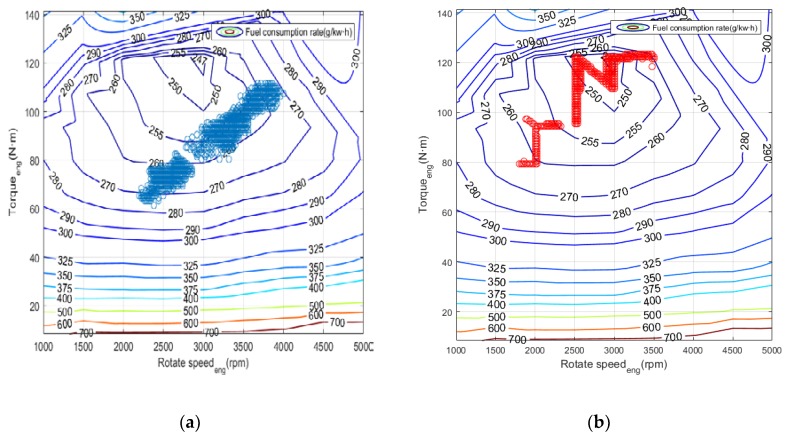
Internal combustion engine operating point distribution: (**a**) Power tracking control; (**b**) Q-Learning control.

In conventional cruise flight missions, the Li-accumulator SOC change curve after using power tracking control and Q-Learning method is shown in Figure 13. It can be found that for the relatively simple task of cruise flight, the SOC curve corresponding to the two control methods has only a slight difference at the end because the required power change frequency is not high. This is due to the cumulative effect caused by different control strategies of the control algorithm. Figure 14 shows the working point distribution of the internal combustion engine of the two control algorithms, the horizontal axis is the speed, the longitudinal axis is the torque, and the contour is the fuel consumption rate. It can be concluded that there is an obvious difference in the working point distribution of the two control modes. Compared with the Q-Learning method, the working point of the power tracking control is more scattered, and the working point of the internal combustion engine obtained by the power tracking control method is distributed in the area with higher fuel consumption rate. Because the working point of Q-Learning only exists in some specific areas, the distribution is more concentrated and more importantly, its working point covers the area with the lowest fuel consumption rate, that is the part less than 250 g/kW·h.

**Figure 15:**
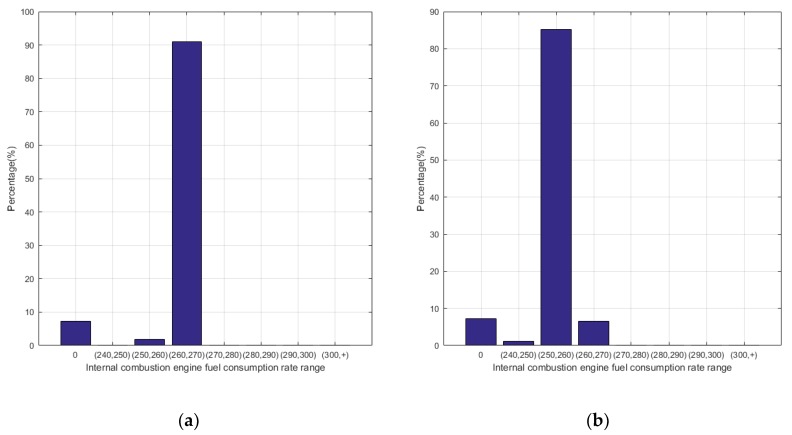
Mathematical Statistics of internal combustion engine operating point. (**a**) Power tracking control; (**b**) Q-Learning control.

**Figure 16:**
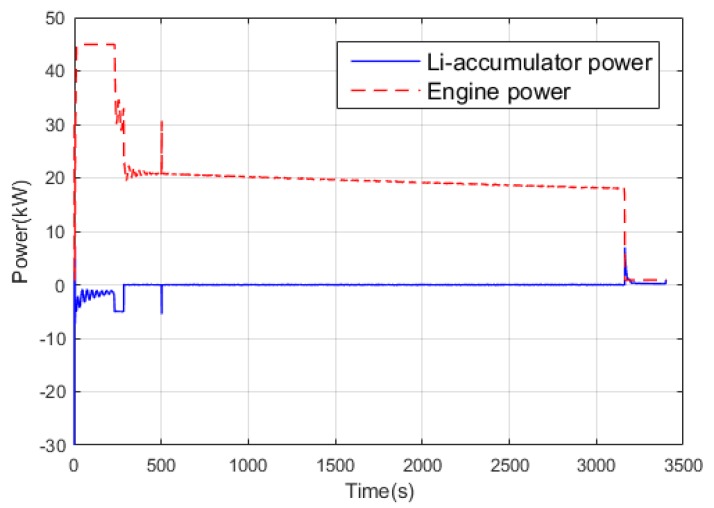
Power variation curve of the Li-accumulator and an internal combustion engine in the power tracking control method.

**Figure 17:**
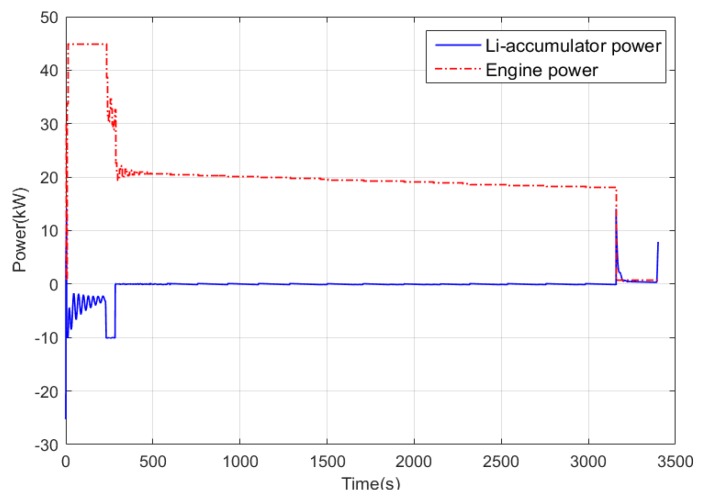
Power variation curve of the Li-accumulator and an internal combustion engine in the Q-Learning control method.

Figure 15 shows the statistics of the operating points of the engine. It can be concluded that for the power tracking control, the working points are mostly concentrated in the interval of (260, 270), while the working points in Q-Learning are mainly concentrated in the range of (250, 260) with lower fuel consumption rate. Figure 16 and Figure 17 show the power distribution of the lithium accumulator and the internal combustion engine. It can be found that the power of the lithium accumulator and the internal combustion engine does not change much in the conventional cruise mission, but there are some ups and downs in the initial stage because they do not reach steady state.

The CO_2_ emission of the aircraft mainly comes from the oxidation of the aviation fuel, and the composition of chemical elements in the aviation fuel determines the fuel CO_2_ emission index. In this paper, the fuel CO_2_ emission index (3150g/kg) proposed by the Intergovernmental Panel on Climate Change (IPCC) [51,52] is used to estimate aircraft CO_2_ emissions. Regardless of engine model, mode of operation, and atmospheric environmental impact, CO_2_ emissions for complete routes can be calculated using the following formula:

(33) $$E_{{\mathrm{CO}}_{2}}=Q_{fuel}\cdot I$$

where $E_{{\mathrm{CO}}_{2}}$ is the total CO_2_ emission of the UAV, $Q_{fuel}$ is the fuel consumption, and I is the fuel emission index (3150 g/kg).

As for the calculation of NO_x_ emissions, this article adopts a classic method of calculating pollution emissions—the Boeing flow method. This method uses the relationship between fuel flow and various pollutant emission indexes in standard ground environmental conditions in the International Civil Aviation Organization (ICAO) emission data to estimate the engine’s pollution emission index [53,54]. Calculated as follows:

(34) $$\begin{array}{c} E_{NO_{x}}=10.148*\frac{W_{fuel}}{\delta }•\theta^{3.8}e^{0.2Ma^{2}}-3.8871\\ \theta =\frac{T}{288.5},\delta =\frac{P}{14.696} \end{array}$$

where $E_{NO_{x}}$ is the total NO_x_ emission of the UAV, $W_{fuel}$ is the actual fuel flow of the engine, θ is the ratio of the engine operating environment temperature to the standard atmospheric temperature, and δ is the ratio of the engine operating environment pressure to the standard ambient pressure and *Ma* is the Mach number when the engine is operating.

As shown in Table 2, we calculated the fuel consumption and CO_2_, NO_x_ emissions of different types of power systems in the conventional cruise flight mission of the UAV according to Equations (33) and (34). At the same time, the parameter indexes of different energy management control algorithms for the same hybrid power system are calculated and compared. The results show that the hybrid power system reduces fuel consumption and CO_2_, NO_x_ pollutant emissions compared to conventional petrol-powered systems. The energy management controller of the hybrid power system designed by the Q-Learning method is better than the one designed by the power tracking control method with a relative reduced rate of at least 2.18%. In a situation where power demand does not change much, such as the conventional cruise flight, since the frequency of the system power supplied by the Li-accumulator is not high, it does not play a significant compensatory effect. The results of both control methods are better than traditional UAV power systems.

#### 4.3.2. Terrain Tracking Flight Mission

**Figure 18:**
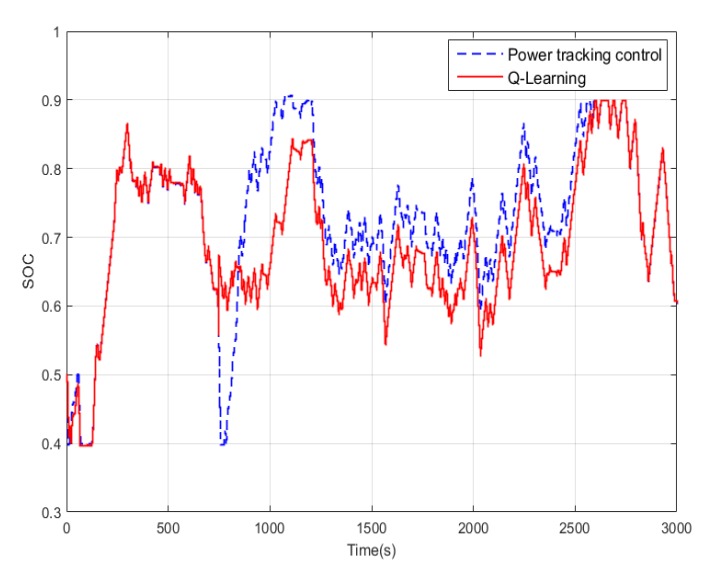
SOC variation curve of two control methods in the terrain tracking flight mission.

**Figure 19:**
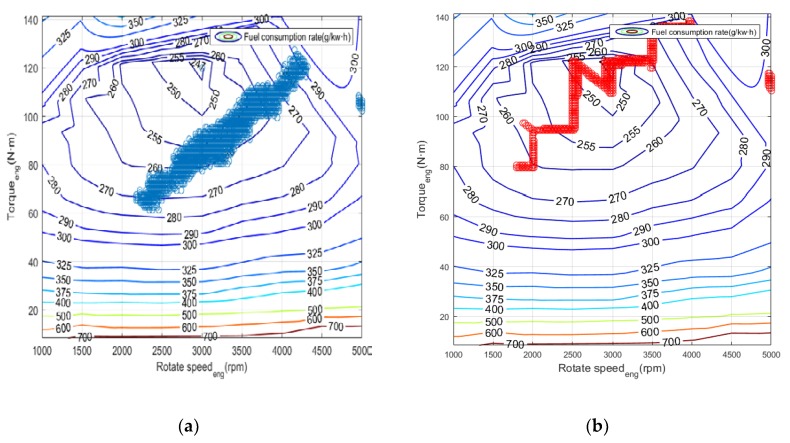
Internal combustion engine operating point distribution. (**a**) Power tracking control; (**b**) Q-Learning control.

**Figure 20:**
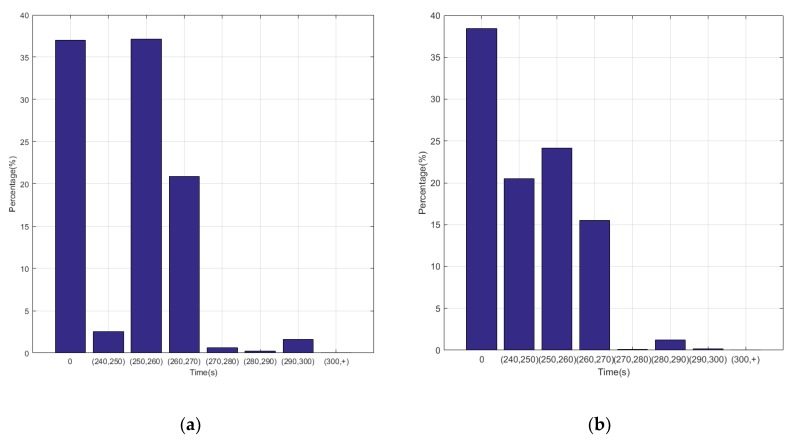
Mathematical statistics of internal combustion engine operating point. (**a**) Power tracking control; (**b**) Q-Learning control.

**Figure 21:**
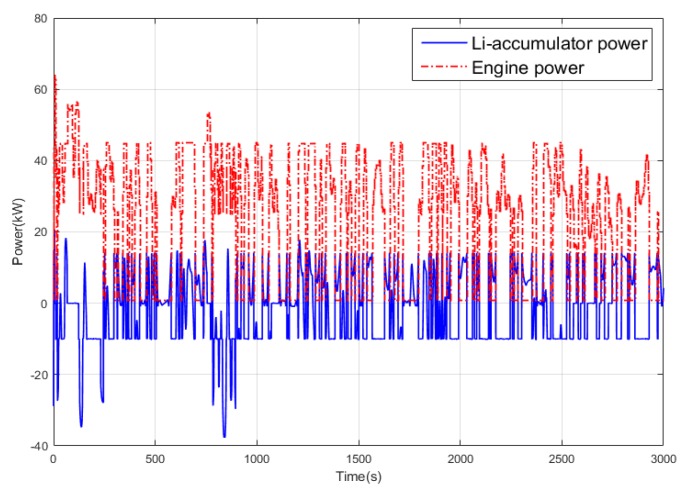
Power variation curve of the Li-accumulator and engine in the power tracking control method.

**Figure 22:**
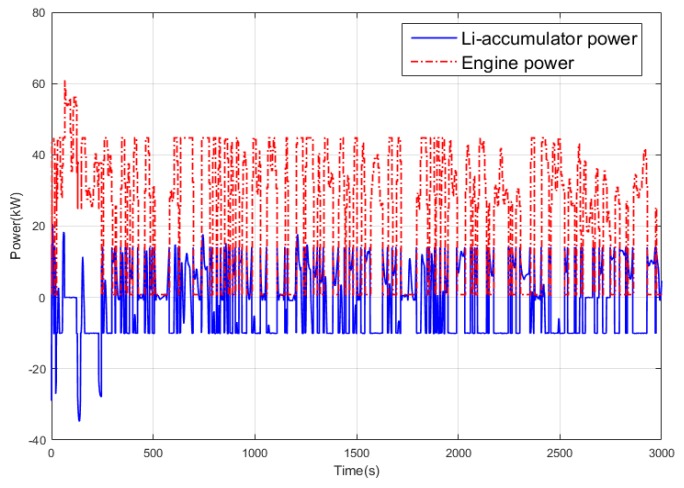
Power variation curve of the Li-accumulator and engine in the Q-Learning control method.

In the terrain tracking mission, UAVs need to track the terrain according to the planned mission route to maintain a certain altitude with the ground. Figure 18 shows that there is a significant difference in the SOC change curve of the Li-accumulator corresponding to the two control methods in the time range (500, 2000). Further analysis of the working point distribution of the internal combustion engine in Figure 19 shows that the working point of the internal combustion engine is more concentrated in the area with lower fuel consumption rate in the result of the Q-Learning control. It is distributed in the nearest area on both sides with the lowest fuel consumption rate as the center, which indicates that the internal combustion engine works more in the efficient area. From the statistical results of the work point distribution in Figure 20, it can be found that there are more work points in the Q-Learning control mode in the low fuel consumption range of (240, 250). In addition, there are many working points in the power tracking control in the areas of (280, 290) and (290, 300) with high fuel consumption, demonstrating that Q-Learning can effectively adjust the power distribution of the internal combustion engine and lithium accumulator and reduce the occurrence of extreme operating points with high fuel consumption. In addition, compared with the conventional cruise flight, it can be found that as a result of a drastic change of power demand in the process of terrain tracking flight, will reduce power demand or even zero power in the flight process of reducing the altitude of the UAV. Therefore, in the terrain tracking mission, the shutdown condition of the internal combustion occurs on more occasions than in the case of the conventional cruise mission. The power distribution of the Li-accumulator and the internal combustion engine in Figure 21 and Figure 22 show that the power distribution mode is obviously different from that of conventional cruise flight because of the frequent change of power demand in the terrain tracking mission. In this case, the Li-accumulator charges and discharges more frequently, the power peaks and filling the power trough of the internal combustion engine, which would have to operate in the area of high consumption, are reduced via engagement of the lithium accumulator to suffice energy demand. This prevents the working condition of leaving the high efficiency area of the internal combustion engine as far as possible.

In the case of different types of power systems and different energy management control algorithms, the fuel consumption and CO_2_, NO_x_ emissions of the UAV in the terrain tracking flight mission are calculated. Comparing to the petrol–electric hybrid system with the traditional UAV power system, it is not difficult to find that a hybrid power system significantly saves the 17.69% aviation fuel of the UAV at least and reduces the emissions of CO_2_, NO_x_ in Table 3. The energy management controller designed by the Q-Learning method is superior to that designed by power tracking control method with a relative decline rate of at least 4.41%. Compared to a controller designed by the power tracking control method, the energy management controller designed by the Q-Learning method reduces the proportion of fuel consumption and pollutant emissions in the terrain tracking mission, which is larger than that of the UAV in the cruise mission. It shows that the control method of Q-Learning is more suitable for the operating conditions where the demand power and flight altitude change frequently.

## 5. Conclusions

In order to focus on the series petrol–electric hybrid propulsion system of the UAV, the subsystem model of the UAV hybrid propulsion system and simulation platform were established based on Matlab/Simulink software and its performance was simulated and analyzed. The energy management controller based on reinforcement learning was designed by using the Q-Learning method and compared with the power tracking control method under the two selected common operating conditions of the conventional cruise and terrain tracking missions. Under these conditions, the hybrid power system UAV significantly reduces fuel consumption in comparison to the traditional petrol-powered UAV. Decrease in fuel consumption leads to the reduction of air pollutants such as CO_2_ and NO_x_, which are linked to be causing the greenhouse effect as well as reducing the stratospheric ozone layer. With less potentially harmful air particles being emitted by the propulsion system, the overall positive impact on public health is represented by mitigating presence of the elements in the air, which were documented to cause severe health issues such as cancer, diabetes, or alteration in the cardiac autonomic function.

Considering energy management algorithms of the hybrid power systems, the Q-Learning method based on reinforcement learning has better control effect on the energy management controller of the hybrid power system, which can obviously improve the working efficiency of the hybrid power system, reduce the fuel consumption of the UAV and emissions of polluting aviation environmental gases compared with the rule-based power tracking control method. The power tracking control method is more suitable for designing the energy management controller of the UAV under the condition that the demanded power changes smoothly. For terrain tracking missions with frequent changes, the fuel consumption saving ratio of the Q-Learning control method is higher, indicating that the reinforcement learning method based on optimization is more suitable for the working conditions with frequent power changes.

## Figures and Tables

**Table 1:** Overall parameters of the UAV.

Parameter Name	Parameter Value
Wing Area (m^2^)	15.24
Aspect Ratio	16.8
Wingspan (m)	16
Length (m)	7.1
Height (m)	1.7
Maximum Take-Off Mass (kg)	770
Bare Weight (kg)	500
Fuel (kg)	80
Propeller Diameter (cm)	160
Thrust-Weight Ratio	0.5
Cruising Speed (m/s)	50
Peng (kW)	50
Pm (kW)	70

**Table 2:** Comparison of parameters and indexes of the UAV during conventional cruise flight mission.

Type	Control Strategy	Fuel/kg	%	CO_2_/kg	%	**NO_x_/kg**	**%**
Petrol Powered UAV	-	11.75	-	37.013	-	0.518	-
Hybrid UAV	Power Tracking	9.965	−15.2	31.391	−15.2	0.3806	−26.52
Hybrid UAV	Q-Learning	9.708	−17.38	30.58	−17.38	0.352	−32.05

**Table 3:** Comparison of parameters and indexes of the UAV terrain tracking flight mission.

Type	Control Strategy	Fuel/kg	%	CO_2_/kg	%	**NO_x_/kg**	**%**
Petrol Powered UAV	-	13.436	-	42.323	-	0.607	-
Hybrid UAV	Power Tracking	11.059	−17.69	34.836	−17.69	0.428	−29.5
Hybrid UAV	Q-Learning	10.467	−22.1	32.97	−22.1	0.395	−34.93
